# *Lactobacillus plantarum* and Galacto-Oligosaccharides Synbiotic Relieve Irritable Bowel Syndrome by Reshaping Gut Microbiota and Attenuating Mast Cell Hyperactivation

**DOI:** 10.3390/nu17101670

**Published:** 2025-05-14

**Authors:** Qi Yao, Wenbo Zhang, Yuze Wang, Le Shi, Yixiao Zhao, Jiarui Liang, Yu Zhao, Jiawei Kang, Xudong Zheng, Rui Guo, Tian Yuan, Yongbo She, Zhigang Liu

**Affiliations:** 1 Laboratory of Functional Chemistry and Nutrition of Food, College of Food Science and Engineering, Northwest A&F University, Xianyang 712100, China; qiyao@nwafu.edu.cn (Q.Y.); zhangwenbo@link.cuhk.edu.hk (W.Z.); wangyz@nwafu.edu.cn (Y.W.); sl2020@nwafu.edu.cn (L.S.); areshinezhao@gmail.com (Y.Z.); jiarui3@nwafu.edu.cn (J.L.); yuzhao029@nwafu.edu.cn (Y.Z.); 15961674680@163.com (J.K.); zhengxudong@nwafu.edu.cn (X.Z.); ruiguo@nwafu.edu.cn (R.G.); 2 Shaanxi Key Laboratory of Natural Products & Chemical Biology, College of Chemistry & Pharmacy, Northwest A&F University, Xianyang 712100, China; tian.yuan@nwafu.edu.cn; 3 Shenzhen Research Institute, Northwest A&F University, Shenzhen 518000, China

**Keywords:** IBS, synbiotic, gut microbiota, inflammation, mast cells

## Abstract

Background: Irritable bowel syndrome (IBS) significantly impairs the lifestyle and quality of life of the global population. However, the underlying pathophysiological mechanisms remain largely elusive. While conventional pharmacological approaches show limited therapeutic efficacy, emerging microbiota-targeted dietary interventions present promising alternatives. Objectives: The present study aimed to elucidate the molecular mechanisms by which a synbiotic mitigates IBS and associated colonic dysfunctions in C57BL/6 mice. Methods: The mouse model was induced by a *Citrobacter rodentium* (*C. rodentium*) infection combined with water avoidance stress (WAS). Galacto-oligosaccharides (GOS) were identified as the optimal carbon source for the growth of *Lactobacillus plantarum* ZYC501 (*L. plantarum* ZYC501), leading to the establishment of the synbiotic formulation. Results: The 32-day synbiotic intervention, consisting of *L. plantarum* ZYC501 (1 × 10^9^ CFU/day) and GOS (10 g/L, *w*/*w*), significantly alleviated colonic transit dysfunction, visceral hypersensitivity, and anxiety-like behaviors in IBS mice. The synbiotic treatment significantly inhibited the expression levels of histamine, mast cell tryptase, and prostaglandin E2 (PGE2) (*p* < 0.05). The synbiotic also suppressed colonic inflammation by reducing the levels of lipopolysaccharide (LPS), *TNF-α*, and *IL-6* (*p* < 0.05). Moreover, the synbiotic increased the expression of MUC2 and the production of short-chain fatty acids (SCFAs), including acetate, propionate, and butyrate (*p* < 0.05). In terms of gut microbiota modulation, the synbiotic reshaped the gut microbiota composition, increasing the abundance of *Lactobacillus* and *Akkermansia* while decreasing the levels of *Helicobacter* and *Saccharibacteria*. Correlation analysis further revealed a strong association among SCFAs, colonic inflammation, and the gut microbiota. Conclusions: In conclusion, the synbiotic composed of *L. plantarum* ZYC501 and GOS effectively alleviates IBS and associated colonic dysfunctions by modulating the gut microbiota, reducing mast cell hyperactivity, and enhancing colonic barrier integrity. These findings provide a theoretical basis for developing gut microbiota-targeted dietary interventions for the management of IBS and improvement in gut health.

## 1. Introduction

Irritable bowel syndrome (IBS) impairs the life quality of approximately 11% of the global population [1]. Diarrhea-predominant IBS (IBS-D) is the most prevalent phenotype and is often characterized by bloating, diarrhea, abdominal pain, intestinal transit dysfunction, and anxiety-depressive behaviors [2]. To date, the mechanisms underlying the exact pathophysiology of IBS still remain inconclusive. Intriguingly, patients with IBS often exhibit significant dysbiosis of gut microbiota [3], compromised gut mucosal barrier integrity [4,5], elevated translocation of endotoxins [6], and increased inflammation [7]. Moreover, the hyperactivation of mast cells was accompanied by elevated expression levels of cyclooxygenase-2 (COX-2). In pre-clinical studies of IBS, prostaglandin E2 (PGE2) has been observed and attributed to, at least in part, lipopolysaccharide (LPS) translocation [8]. The elevation of PGE2 levels is intricately linked to visceral hypersensitivity in the context of IBS [9,10]. Recent studies have further revealed that mechanisms of IBS are closely linked to dysfunction of the gut barrier and disruption of gut microbiota [11,12,13]. Therefore, focusing on the regulation of gut microbiota may potentially serve as a pivotal strategy for alleviating IBS.

Conventionally, administration of medications such as otilonium and loperamide is considered to be the primary pharmacological approach for managing IBS, yet they are often associated with concerns about a range of side effects, including drowsiness, fatigue, and some safety concerns [14,15]. In contrast, lifestyle and/or dietary manipulations may be more effective and safer alternatives, including the recommendation for selecting a low FODMAPs (low fermentable oligosaccharides, disaccharides, monosaccharides, and polyols) diet, supplementing probiotics as well as prebiotics [16,17]. However, despite the fact that a low FODMAP diet ought to be consumed with less fermentable carbohydrates and thus avoid excessive abdominal discomfort, these oligosaccharides can also serve as prebiotics, modulating gut microbiota diversity, and overall, the gut health [18,19].

Probiotics have emerged as an effective intervention targeting the modulation of gut microbiota in the context of IBS [20,21]. Notably, specific strains such as *Lactobacillus rhamnosus* GG and *Bifidobacterium bifidum* MIMBb75 have been demonstrated clinically in alleviating diarrhea and abdominal pain in pediatric IBS populations [16,22]. Furthermore, accumulating pre-clinical and clinical evidence demonstrated that *Lactobacillus plantarum* ameliorates the hallmark manifestations of IBS, including diarrhea, abdominal discomfort, inflammation, as well as dysregulated gut microbiota [23,24,25,26]. These findings collectively position *Lactobacillus plantarum* as a promising probiotic for managing IBS and its associated gastrointestinal perturbations. On the other hand, prebiotics such as Galacto-oligosaccharides (GOS), fructo-oligosaccharides (FOS), xylo-oligosaccharides (XOS), and inulin have also been shown to have similar effects as probiotics, including the mitigation of abdominal symptoms and the modulation of the composition of the gut microbiota [27,28], elevation of short-chain fatty acids (SCFAs) levels, and enhancement of gut barrier integrity [29,30]. Collectively, these pieces of evidence suggest that targeting gut microbiota would be a useful approach to alleviate IBS and its associated gut dysfunctions. However, there are certain drawbacks associated with the efficacy that rely solely on pro- and/or prebiotics, including a low colonization efficiency of probiotics in the colon [31]. Thus, consider the fact that an “optimal” carbon source may be utilized to maximize energy production and thereby facilitate the growth and colonization of probiotics in the gut [32,33]. An appropriate synbiotic reflects an excellent means of establishing an effective strategy that may counteract intestinal dysfunctions, particularly in the context of IBS [34,35].

In the present study, the aim was to elucidate the mechanisms by which synbiotics mitigate IBS and its associated intestinal dysfunction in a mouse model induced by *Citrobacter rodentium* (*C. rodentium*) and WAS (chronic water avoidance stress). Firstly, *Lactobacillus plantarum* ZYC501 (strain number: GDMCC 64356, *L. plantarum* ZYC501) was screened and identified from the feces of healthy individuals. Subsequently, a synbiotic formulation was developed through the combination of *L. plantarum* ZYC501 and GOS based on a growth curve experiment. In the animal experiment, *L. plantarum* ZYC501 was administered to the IBS + *L. p.* group, GOS was administered to the IBS + GOS group, and the synbiotics of *L. plantarum* ZYC501 and GOS were administered to the IBS + SYN group. Subsequently, a series of behavioral tests was conducted to assess the beneficial effects of the synbiotics on colonic transit function, visceral hypersensitivity, and anxiety-like behavior. To further delineate the underlying mechanisms, mucosal barrier integrity, colonic inflammation, gut microbiota composition, and microbial metabolites were determined, accompanied by correlation analyses with biochemical markers and mast cell hyperactivation. The findings from the present study provide new evidence for exploring the mechanism of synbiotics to alleviate IBS and lay a foundation for alleviating IBS through non-pharmacological interventions.

## 2. Materials and Methods

### 2.1. Animals

Male eight-week-old C57BL/6 mice were purchased from Xi’an Jiaotong University. All experimental animals were housed within a temperature-controlled vivarium at Northwest A&F University under standardized environmental conditions (22 ± 2 °C, 55 ± 5% relative humidity) and a regulated 12 h photoperiod (light/dark cycle) to ensure physiological consistency and compliance with institutional ethical guidelines. All animals were maintained in individual housing with ad libitum access to a nutritionally standardized diet (AIN-93M purified formulation; Trophic Animal Feed High-Tech Co., Ltd., Nantong, China) and autoclaved water to minimize environmental variability and ensure metabolic stability throughout the experimental period. The complete nutritional profile of the AIN-93M purified diet is comprehensively detailed in Table S1. The animal experimental protocols were adhered to from the 8th edition of the Guide for the Care and Use of Laboratory Animals [36] and received full institutional review and approval from the Animal Ethics Committee of Northwest A&F University, Yangling, China (Ethics Approval No. XN2023-0712, 12 July 2023).

### 2.2. Probiotic Growth Curve

The genomic sequence of *L. plantarum* ZYC501 (strain number: GDMCC 64356) was determined through whole-genome sequencing, as previously described [37]. Phylogenetic alignment analysis against the CAZy (carbohydrate-active enzymes) database enabled a systematic annotation of the carbohydrate-active enzymes encoded within the organism’s genome. Substrate specificity screening was subsequently applied to prioritize glycoside hydrolases, demonstrating catalytic competence toward the target carbon substrate, which facilitated in silico prediction of strain-specific prebiotic candidates.

A total of 30 μL of the *L. plantarum* ZYC501 bacterial suspension (1 × 10^11^ CFU/mL) was aseptically inoculated into each well and analyzed by Bioscreen-C (PerkinElmer, Co., Ltd., Turku, Finland), configured to accommodate standard 100-well assay plates, to enable high-throughput monitoring of microbial proliferation dynamics.

After that, 300 μL of carbon sources, including GOS, FOS, XOS, and inulin (Solarbio Science & Technology Co., Ltd., Beijing, China) at various concentrations (5 g/L, 10 g/L, 20 g/L, and 30 g/L) were introduced into the experimental system, while the Gifu Anaerobic Medium (GAM, Qingdao Haibo Biotechnology Co., Ltd., Qingdao, China), devoid of exogenous carbon substrates, served as the negative control. Bacterial proliferation kinetics in media supplemented with strain-specific prebiotic candidates were quantified via real-time optical density monitoring using the Bioscreen-C system over a 48h incubation period.

### 2.3. Animal Experiment Procedure

At the age of 8 weeks, all mice were randomly divided into the following five groups, and each group contained 10 mice: the CON group, IBS group, IBS + *L. p.* group, IBS + GOS group, and IBS + SYN (synbiotic) group. The experiment was conducted as described [38], but with modifications [24]. The intervention period for each group was 33 days. Briefly, on Day 1 of the experiment, the mice in the CON group were administered 200 μL of sterile saline via gavage, whereas the remaining mice were administered *C. rodentium* (ATCC10006) (1.2 × 10^10^ CFU/200 μL) via gavage. From Day 2 to Day 8, to safeguard against the onset of dehydration caused by diarrhea, which could potentially confound the experimental outcomes and compromise the health of the animals, all the mice in the study were administered a subcutaneous injection of 0.5 mL of lactated Ringer’s solution (Qingdao Jisskang Biotechnology Co., Ltd., Qingdao, China). From Day 18 to Day 33 of the experiment, all mice in the IBS group were exposed to WAS to induce chronic psychological stress. Mice in the CON group were positioned on a platform for a duration of 1 h, and during this time, there was no water available to them. After a daily stress induction, mice were administered PBS (CON, IBS, 200 μL) via oral gavage, *L. plantarum* ZYC501 (IBS + *L. p.*, IBS + SYN, 1 × 10^9^ CFU/d) via oral gavage, GOS in the drinking water (IBS + GOS, IBS + SYN, 10 g/L) and for 32 continuous days. To minimize the stress associated with repeated gavage procedures, we opted to provide the prebiotic through the drinking water, thereby reducing the overall stress and ensuring a more reliable and sustainable experimental setup. From Day 30 to Day 33, behavioral tests were conducted.

### 2.4. Behavioral Assessments

#### 2.4.1. Open Field Test

The open field test (OFT) is commonly employed to quantitatively assess anxiety-related behaviors and spontaneous locomotor activity in mice, as delineated in the established ethological protocols previously [39]. The OFT was executed in a 40 × 40 × 40 cm white open-top arena partitioned into a central (24 × 24 cm) and peripheral zone. A digital camera was centrally mounted above the arena within a well-illuminated behavioral testing facility. Ten mice per experimental group were centrally positioned in the arena for 5-min spontaneous locomotion trials. The testing area was further categorized into a central domain and four corner sectors. A computerized video-tracking system (Shanghai Xinruan Information Technology Co., Ltd., Shanghai, China) was utilized to quantify the total distance traveled, central zone distance, and central zone occupancy time, thereby assessing anxiety-like behavior in novel environments.

#### 2.4.2. Marble Burying Test

The marble burying test (MBT) is a widely validated behavioral paradigm for assessing anxiety-like behaviors of mice, as described previously [40]. For the MBT, test cages were filled with a 5 cm thick layer of wood chip bedding. Twenty 15 mm diameter glass marbles were arranged in a 4 × 5 grid on the bedding. Ten mice per group were placed in individual cages, and the number of marbles buried to two-thirds of their height within 30 min was quantified.

#### 2.4.3. Physiological Markers of Colonic Content Transit

As previously described [4], colonic transit time was used to evaluate colonic transit function. After a 32-day intervention, ten mice per cohort were individually housed for 12-h acclimation before colonic transit assessment. Aseptically, 0.15 mL of saturated carmine red suspension was orally administered. Fecal monitoring was initiated 2 h post-gavage; blinded investigators collected fecal pellets every 15 min or upon defecation. The time until the first carmine-stained feces, a validated marker in murine colonic motility studies, was determined as the colonic transit time.

#### 2.4.4. Visceral Hypersensitivity

The assessment of visceral hypersensitivity, which was induced by CRD (colorectal distention), was carried out by employing the abdominal withdrawal reflex (AWR) method, following the protocol that had been previously reported in reference [24]. In the preparation phase of the AWR experiment, the mice (*n* = 10 per cohort) were subjected to a fasting period of 16 h. During the actual testing process, the mice were carefully maintained under isoflurane anesthesia. And then, the air pressure increased gradually to 100, 200, and 300 μL.

### 2.5. Real-Time qPCR

To measure the mRNA expression levels of the genes regulating inflammatory cytokines and proteins, total RNA was isolated from colon tissues with the BIOZOL reagent (Hangzhou Bioer Technology Co., Hangzhou, China) according to the manufacturer’s instructions. Murine-specific primers (Table S2) were employed for qRT-PCR amplification. The Ct values were normalized to the endogenous control gene GAPDH, and relative transcript abundance was calculated using the 2^−ΔΔCt^ method.

### 2.6. Determination of Biochemical Indicators

ELISA was used to measure LPS, histamine, mast cells tryptase, and PGE2 (Mouse Prostaglandin E2 ELISA Kit, Elabscience Biotechnology Co., Ltd., Wuhan, China; Mouse LPS Elisa kit, Mouse Histamine Elisa kit, Mouse Mast cells trypatase Elisa kit, Fankewei Biology Technology, Shanghai, China).

### 2.7. The H&E and Alcian Blue Staining

According to the previously published reference [40], euthanasia was performed under terminal anesthesia via cervical dislocation, followed by immediate dissection and collection of intestinal tissues for subsequent histopathological and molecular analyses. Samples were embedded in paraffin and sectioned (4 μm), then stained with a hematoxylin and eosin staining kit (Beijing Solarbio Science & Technology Co., Ltd., Beijing, China). The H&E staining was entrusted to a company (Wuhan Servicebio Biotechnology Co., Ltd., Wuhan, China) for processing. Sections were evaluated based on the cell infiltration of inflammatory cells and epithelial damage. For alcian blue staining, this was according to the Alcian Blue Stain Kit manufacturer’s instructions (Solarbio Science & Technology Co., Ltd., Beijing, China), as previously described [41]. After the dewaxing of tissue sections, alcian blue/nuclear-fast red was used for 10−20 min and then washed with distilled water, while nuclear solid red staining solution was dyed for 5−10 min and then washed with distilled water to observe the mucus layer and count the goblet cells. The histological changes and alcian blue staining were observed with optical microscopy (Olympus, Tokyo, Japan).

### 2.8. Immunofluorescence Staining

MUC2 and COX-2 were subjected to immunofluorescence staining [42]. Colon tissue was sectioned at 4 μm and paraffin-embedded. Samples were deparaffinized with dimethylbenzene and rehydrated through an alcohol series. Permeabilization was achieved using 0.5% Triton-X 100 for 15 min, followed by antigen retrieval in citrate buffer via heat treatment. Sections were blocked with 3% H_2_O_2_ to quench the endogenous peroxidase activity and then with goat serum prior to incubation with the following targeted primary antibodies, MUC2 (EPR23479-47) (1:200, Cell Signaling Technology, Inc., Beverly, MA, USA) and COX-2 (ET1610-23) (1:200, Hangzhou Huaan Biotechnology Co., Ltd., Hangzhou, China) at 4 °C overnight. Then, these were washed thrice with PBS and incubated with Alexa Fluor-conjugated anti-rabbit secondary antibody and Cy3 conjugated Goat Anti-Rabbit IgG (1:200, Wuhan Servicebio Biotechnology Co., Ltd., Wuhan, China) at 37 °C for 20 min. The immunofluorescence images were evaluated on an inverted fluorescent microscope (Olympus, Tokyo, Japan).

### 2.9. 16S rRNA Sequencing Analysis

Fecal samples were collected in a sterile environment. Then, total cellular DNA was extracted from these samples using the E.Z.N.A. Stool DNA Kit (Omega, Norcross, GA, USA) strictly following the manufacturer’s instructions. The bacterial hypervariable V3-V4 region of 16S rRNA was chosen for the MiSeq (Illumina, San Diego, CA, USA) paired-end 300 bp amplicon analysis using primers 341_F (5′-CCTACGGGNGGCWGCAG-3′) and 802_R (5′-TACNVGGGTATCTAATCC-3′). Library preparation and species difference analyses were carried out according to a previously reported protocol [43]. The fragment size distribution and library concentration were evaluated using the Agilent 2100 Bioanalyzer (Agilent Technologies, Santa Clara, CA, USA). Subsequently, eligible libraries were sequenced on the HiSeq platform, with the sequencing strategy tailored to the insert fragment lengths. The obtained filtered Clean Data served as the basis for downstream analyses. Sequence assembly was accomplished using FLASH software (v1.2.11), which joined paired-end reads through overlapping sequences to generate Tags corresponding to the hypervariable regions. These Tags were then clustered into Operational Taxonomic Units (OTUs) via USEARCH software (v7.0.1090). The OTUs were annotated by comparing them against a reference database for species identification. Species difference analyses between groups were conducted via the Kruskal–Wallis test based on the OTU and annotation results. The Venn Diagram package in R (v3.1.1) was harnessed to visualize overlapping taxa, while Partial Least Squares Discriminant Analysis (PLS-DA) was performed using the mixOmics package in R (v3.2.1).

### 2.10. Quantification of SCFAs Levels

The concentration of SCFAs in the feces sample was determined through the utilization of a gas chromatograph (GC), as previously described [44]. First, combine 200 mg of the fecal sample with 1.6 mL of distilled water, 200 μL of a 50% H_2_SO_4_ solution, and 2 mL of diethyl ether. Place the mixture on ice and shake it forcefully for 20 min. After that, centrifuge the sample at 12,000 rpm for 5 min. Next, carefully transfer the upper diethyl ether layer through a 0.22 μM filter membrane into a new centrifuge tube. Then, use nitrogen gas to blow the solution down to a volume of 200 μL. Finally, transfer the resulting solution to a vial for analysis using GC. GC was performed under the following conditions: a DB-FFAP capillary column (30 cm × 0.25 μm × 0.25 mm) (Agilent Technologies, Wilmington, DE, USA) was used in conjunction with a hydrogen flame detector. The injection port was maintained at 250 °C, and the detector temperature was set at 270 °C. A 2 μL sample was injected in split mode with a split ratio of 10:1 and a nitrogen carrier gas flow rate of 2 mL/min. The temperature program consisted of an initial hold at 50 °C for 10 min, followed by a ramp of 15 °C/min to 120 °C, then 5 °C/min to 170 °C, another ramp of 15 °C/min to 220 °C, and a final hold at 220 °C for 5 min.

### 2.11. Correlation Analysis

The correlation analysis was conducted in accordance with previously published research [45]. Pearson’s correlation coefficients were employed to measure and quantify the interdependence among the relative abundance of the represent microbiome, SCFAs, and IBS-alleviating effects of the synbiotics. The data related to the relative abundance of the represent microbiome, SCFAs, and IBS-alleviating effects of the synbiotics of CON, IBS, and IBS + SYN (*n* = 6) were analyzed on the Weishengxin Visualization Platform.

### 2.12. Statistical Analysis

Data were presented as mean ± SEM from a minimum of three independent experimental replicates. Statistical analysis for multiple comparisons was executed using Tukey’s test, facilitated by GraphPad Prism 9.5 software. Means with different letters (a, b, c, d) are significantly different from each other (*p <* 0.05).

## 3. Results

### 3.1. Establishing a Synbiotic of L. plantarum ZYC501 and GOS

**Figure 1:**
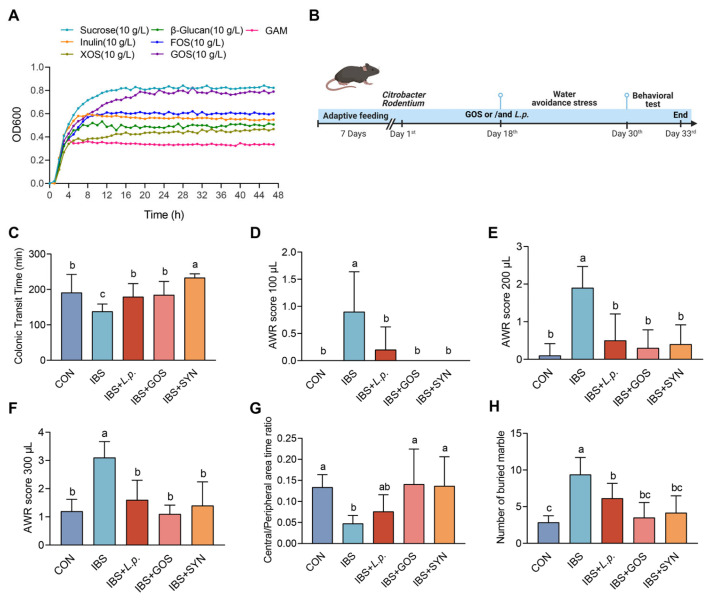
The effects of the synbiotic on the behavioral assessments. (**A**) Growth curves of *L. plantarum* ZYC501 at 10 g/L sucrose, FOS, inulin, GOS, XOS, glucose, and GAM. (**B**) Schematic of the treatment with GOS and/or *L. plantarum* ZYC501. (**C**) Colonic transit function (*n* = 10). (**D**–**F**) Comparison of the AWR scores of different groups. Air pressures of 100 μL, 200 μL, and 300 μL (*n* = 10). (**G**) Central area time/ peripheral area time ratio in the open field test (*n* = 10). (**H**) Number of buried marbles (*n* = 10). Means with different letters (a, b, c) are significantly different from each other (*p <* 0.05).

CAZy analysis of the whole-genome sequencing data obtained for *L. plantarum* ZYC501 uncovered the presence of specific enzymes within its genetic repertoire. These enzymes are crucial for the bacterium’s ability to effectively hydrolyze GOS and FOS; the detailed protocol of CAZy was previously described [43]. *L. plantarum* ZYC501 exhibited the optimal growth trend in media supplemented with GOS compared to other carbon sources, including FOS, XOS, and inulin, at various concentrations (Figure 1A and Figure S1). These findings suggest that GOS is the optimal prebiotic for *L. plantarum* ZYC501, thereby establishing a strain-specific synbiotic formulation.

### 3.2. The Synbiotic Alleviated Colonic Transit Dysfunction, Visceral Hypersensitivity, and Anxiety-like Behaviors in IBS Mice

The progression of IBS-D is frequently associated with abnormal colonic transit function and visceral hypersensitivity, accompanied by the emergence of anxiety-like symptoms [46,47]. Eight-week-old male C57BL/6 mice were exposed to *C. rodentium* and WAS, followed by treatment with either PBS, GOS, *L. plantarum* ZYC501, or a synbiotic treatment (Figure 1B). IBS mice showed a significant reduction in colonic transit time compared to the CON mice. Mice in all intervention groups showed an improved colonic transit time compared to the IBS group, while the colonic transit time in IBS + SYN was significantly longer than all other groups (Figure 1C). The visceral hypersensitivity of the mice was assessed through CRD and reflected by AWR. Under the stress conditions (100, 200, and 300 μL), mice in the IBS group exhibited significant abdominal contractions and arched posture compared to the CON, whereas the AWR scores in all intervention groups were significantly lower than in the IBS group and alleviated to a similar level as the CON (Figure 1D–F). Furthermore, to determine the alleviation effects of the synbiotic on the anxiety-like behaviors in the context of IBS, OFT was conducted. As expected, the IBS mice exhibited a significantly lower central/peripheral area time ratio and more buried marbles compared to the CON group (Figure 1G,H), suggesting anxiety-like behaviors. In contrast, mice in all intervention groups improved the central/peripheral area time ratio and decreased the number of buried marbles to a similar level as the CON group, notably IBS + GOS and IBS + SYN exhibited a greater effect on alleviating anxiety-like behaviors compared to IBS + *L. p.* Collectively, these results indicated that the model of IBS was successfully established and the intervention including *L. plantarum* ZYC501, GOS, and the synbiotic alleviated colonic transit dysfunction, visceral hypersensitivity, and anxiety-like behaviors in IBS mice.

### 3.3. Effects of Synbiotic Treatment on Modulating Mast Cell Hyperactivation

**Figure 2:**
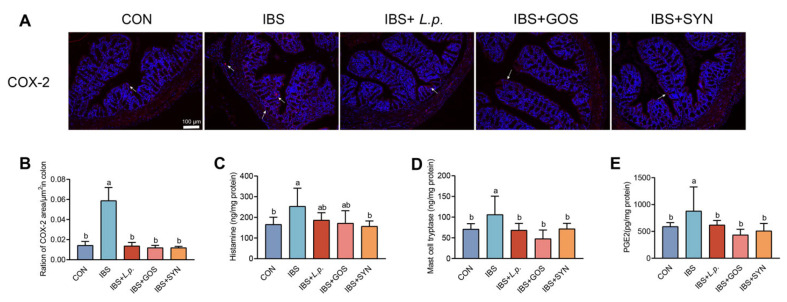
Effect of the synbiotic on mast cell hyperactivation. (**A**) Representative immunohistochemical-stained images of COX-2 expression in the colon. (*n* = 3). The expression sites of the COX-2 were marked by the white arrows. Scale bars: 100 µm. (**B**) Quantification of COX-2 area based on immunohistochemical staining by ImageJ 1.52a software (*n* = 3). (**C**) Levels of histamine in the colon (ng/mg protein) (*n* = 6). (**D**) Levels of mast cell tryptase in the colon (ng/mg protein) (*n* = 6). (**E**) Levels of PGE2 in the colon (ng/mg protein) (*n* = 6). Means with different letters (a, b) are significantly different from each other (*p <* 0.05).

To assess mast cell hyperactivation, the key biomarkers, including histamine, mast cell tryptase, and PGE2, were determined by an ELISA kit in the colonic tissue of the mice. The results demonstrated that histamine, mast cell tryptase, and PGE2 levels in the IBS group were significantly elevated compared to the CON group. Furthermore, following a series of interventions including *L. plantarum* ZYC501, GOS, and synbiotics, histamine, mast cell tryptase, and PGE2 levels showed a significant restoration (Figure 2C–E). The expression of COX-2 in the colon was assessed by immunofluorescence staining (Figure 2A). The findings revealed that the expression of COX-2 in the colonic tissue in the IBS group was significantly elevated compared to the CON group, while interventions with *L. plantarum* ZYC501, GOS, and the synbiotic effectively diminished the COX-2 levels (Figure 2B). Thus, all interventions were able to restore the biomarker levels to normal levels; however, only the synbiotic, rather than *L. plantarum* ZYC501 and GOS, reduced the histamine levels. These results revealed that IBS mice exhibited significant mast cell hyperactivation, while the synbiotic effectively reduced mast cell hyperactivation.

### 3.4. Synbiotic Treatment Enhances the Integrity of the Colonic Barrier in IBS Mice

**Figure 3:**
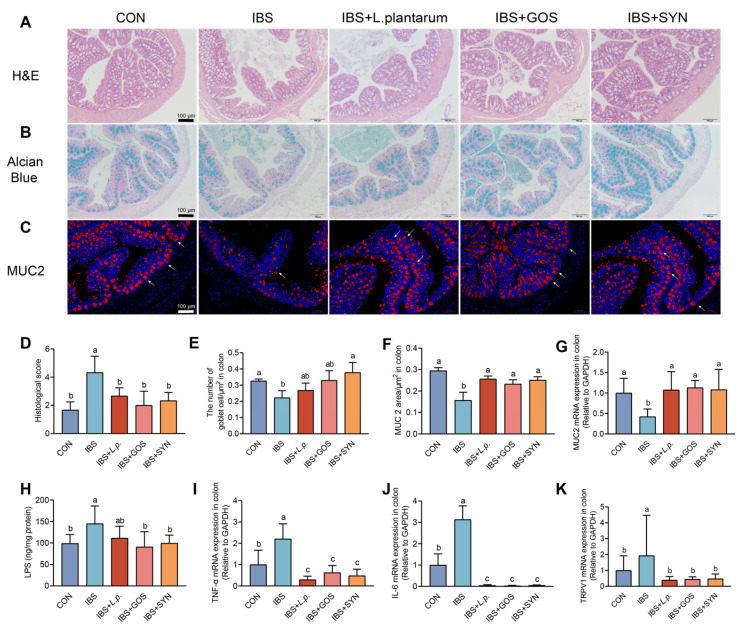
Synbiotic treatment alleviated colonic barrier integrity and inflammation in IBS mice. (**A**) Representative H&E-stained images of the colon tissue. Scale bars: 100 µm. (**B**) Representative Alcian blue-stained images of the colon. Scale bars: 100 µm. (**C**) Representative immunohistochemical-stained images of MUC2 expression in the colon. The expression sites of the MUC2 were marked by the white arrows. Scale bars: 100 µm. (**D**) Histopathological scores (*n* = 3). (**E**) Quantification of goblet cells’ area based on Alcian blue staining by ImageJ software (*n* = 3). (**F**) Quantification of MUC2 area based on immunohistochemical staining by ImageJ software (*n* = 3). (**G**) The mRNA levels of *MUC2* in the colon (*n* = 6). (**H**) Levels of LPS in the colon (ng/mg protein) (*n* = 6). (**I**–**K**) The mRNA levels of *TNF-α*, *IL-6*, and *TRPV1* in the colon (*n* = 6). Means with different letters (a, b, c) are significantly different from each other (*p <* 0.05).

To determine the pathological alterations in the colonic tissue of the IBS mice and the alleviation effects of the synbiotic, mucosal integrity and goblet cells were assessed by H&E and Alcian blue staining. As demonstrated in Figure 3A,B, the histological scores of the IBS group were significantly higher than in the CON group (Figure 3D), suggesting decreased muscle thickness, crypt damage, and impaired mucosal integrity. In contrast, mice in all the intervention groups showed significant improvement in the histological scores compared to the mice in the IBS group. Similarly, Alcian blue staining revealed a reduction in the number of goblet cells in the colonic tissue of IBS mice compared to the control mice (Figure 3E). Regardless of the interventions, the number of goblet cells was improved to a similar level to the CON, while the number of goblet cells in IBS + SYN was significantly higher than in the IBS group. Furthermore, immunofluorescence staining was employed to evaluate the expression of MUC2 in the colon (Figure 3C). As expected, the expression of MUC2 in the IBS group was significantly lower than that in the CON group. Mice in all intervention groups showed improved MUC2 expression levels (Figure 3F), and the mRNA levels (Figure 3G) were significantly higher than in the IBS group. Collectively, these results suggested that the integrity of the gut barrier in IBS mice was compromised, and the intervention of *L. plantarum* ZYC501, GOS, and the synbiotic can restore gut barrier integrity by increasing the number of goblet cells and expression of MUC2 in the colon.

### 3.5. Synbiotic Treatment Effectively Reduces Colonic Inflammation in the IBS Mice

The expression levels of inflammatory factors at the mRNA level within colonic tissue were assessed by using qRT-PCR. Additionally, the concentration of LPS in relevant samples, derived from the colonic tissue, was determined through the application of an ELISA. As expected, the LPS levels in the IBS group were significantly higher than in the CON, while the intervention of GOS and the synbiotic, but not *L. plantarum* ZYC501, reduced the LPS levels (Figure 3H). The mRNA levels of *TNF-α* and *IL-6* were examined in colonic tissue, and similarly, *TNF-α* and *IL-6* mRNA levels were significantly higher in the IBS group than in the CON group, whereas the intervention of *L. plantarum* ZYC501, GOS, and synbiotics reduced the *TNF-α* and *IL-6* mRNA levels (Figure 3I,J). Similarly, the mRNA expression levels of *TRPV1* (Transient receptor potential vanilloid 1) in the IBS group were higher than those observed in both the CON and all intervention groups (Figure 3K). These results suggested that inflammatory responses and *TRPV1* in the IBS group and the capacity of the interventions with *L. plantarum* ZYC501, GOS, and the synbiotic mitigate these inflammatory responses and *TRPV1*.

### 3.6. Effects of Synbiotic Treatment on SCFAs Production and Gut Microbiota Diversity in IBS Mice

**Figure 4:**
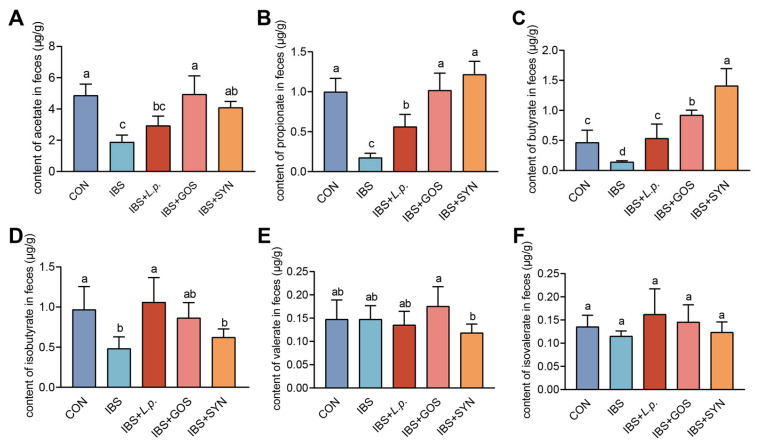
Effects of synbiotic on SCFAs in the feces of IBS mice. The level of SCFAs in the feces (μg/g): (**A**–**F**) acetate; propionate; butyrate; isobutyrate; valerate; isovalerate (*n* = 6). Means with different letters (a, b, c) are significantly different from each other (*p <* 0.05).

SCFAs in fecal samples from mice were quantified by GC. The result of the SCFAs indicated that the levels of acetate, propionate, and butyrate were decreased in the IBS group compared to the CON group (Figure 4A–C). Following the intervention of *L. plantarum* ZYC501, GOS, and the synbiotic, the levels of acetate, propionate, and butyrate in the feces exhibited a significant increase compared to the IBS group (Figure 4A–C). Notably, both GOS and the synbiotic elicited significantly elevated propionate synthesis compared to *L. plantarum* ZYC501. Furthermore, the synbiotic cohort demonstrated an increase in butyrate production relative to both the IBS + *L. p.* and IBS + GOS groups. These results demonstrated that the supplement of *L. plantarum* ZYC501, GOS, and the synbiotic could effectively regulate the contents of the metabolites of gut microbiota, further suggesting the metabolic advantage of synbiotics to modulate SCFAs.

**Figure 5:**
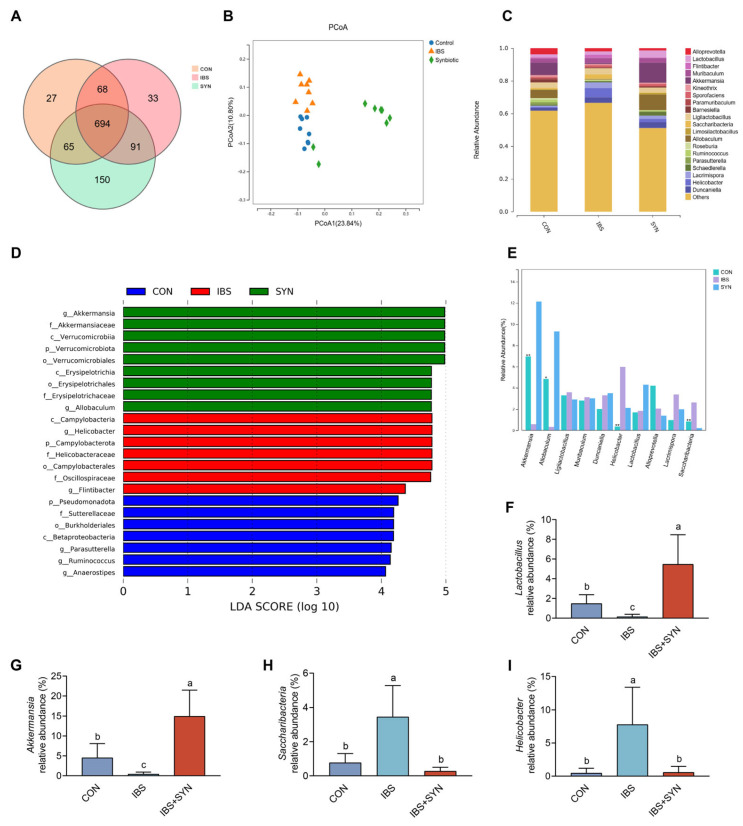
Synbiotic modulated gut microbiota diversity in the IBS mice. (**A**) Venn diagram of the CON, IBS, and SYN groups. (**B**) PCoA of groups. (**C**) Representative differentially abundant gut microbiota at the genus level among the CON, IBS, and SYN groups. (**D**) A display diagram of significantly different species with an LDA score greater than 4.0 (*n* = 6). (**E**) Genus-level microbial diversity analysis of the CON, IBS, and SYN groups. Data presented as mean ± SEM * *p* < 0.05, ** *p* < 0.01 versus the CON group. (**F**–**I**) Relative abundance of *Lactobacillus*, *Akkermansia*, *Helicobacteraceae*, and *Saccharibacteria* at the genus level (*n* = 6). Means with different letters (a, b, c) are significantly different from each other (*p <* 0.05).

To evaluate the effect of synbiotic intervention on the diversity of the gut microbiota in mice with IBS. The fecal samples were analyzed through 16S rRNA gene sequencing. According to the Venn diagram analysis results, 854 OTUs were identified in the CON group, 886 OTUs were detected in the IBS group, and as many as 1000 OTUs were found in the IBS + SYN group. Significantly, the CON group had 27 exclusive OTUs. The IBS group contained 33 unique OTUs, and the IBS + SYN group boasted 150 distinct OTUs (Figure 5A). Principal coordinates analysis (PCoA) illustrated that the samples predominantly clustered according to treatment of synbiotics (Figure 5B), indicating significant alterations in the composition of the gut microbiota among the CON, IBS, and IBS + SYN groups. The IBS group demonstrated a significant reduction in gut microbiota diversity compared to the CON group. In an effort to comprehensively understand the differences in the microbial communities among the distinct experimental groups, linear discriminant analysis was utilized. Notably, the IBS group exhibited a marked decrease in the genera *Akkermansia*, *Allobaculum*, and others compared to the CON group. However, the IBS group showed a significant increase in the genera Helicobacter, Flintibacter, and others compared to the CON and IBS + SYN groups (Figure 5D). An analysis of microbial diversity uncovered substantial modifications at the genus-level taxonomic rank, suggesting that synbiotic intervention reshaped the gut microbiota by increasing the abundance of *Lactobacillus* and *Akkermansia* (Figure 5C,E–G) while decreasing the levels of *Helicobacter* and *Saccharibacteria* (Figure 5C,E,H,I). These findings suggested that the gut microbiota composition exhibited significant alterations in the IBS group compared to the CON group, and synbiotics effectively modified the composition of the gut microbiota in IBS mice.

### 3.7. Correlational Analyses

**Figure 6:**
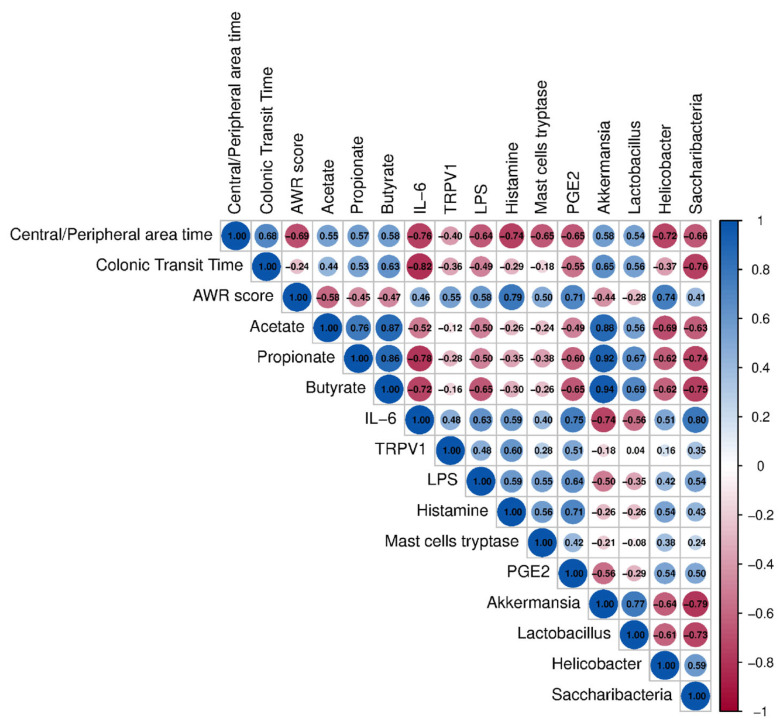
Correlation analysis among behavior, the level of SCFAs, and gut microbiota. In the upper right quadrant of the figure, the diameter of the circles signified the magnitude of the correlation index. The size and color of the circles indicate the degree of correlation: red denoted a positive correlation, while blue indicated a negative correlation (*n* = 6).

To explore the correlation among the gut microbiota composition, SCFA concentrations, and other parameters related to the gut barrier function and inflammatory responses in IBS mice, Pearson’s correlation analysis was performed using the experimentally derived parameters (Figure 6). The relative abundances of *Akkermansia* (r = 0.65) and *Lactobacillus* (r = 0.56) exhibited positive correlations with the discrimination index in colonic transit time. Furthermore, the analysis revealed that the relative abundance of *Akkermansia* was positively correlated with fecal levels of acetate (r = 0.88), propionate (r = 0.92), and butyrate (r = 0.94) in the IBS mice. Moreover, an intriguing result was observed: *IL-6* exhibited a significant positive correlation with the AWR scores (r= 0.46), the expression of *TRPV1* (r = 0.48), LPS levels (r = 0.63), histamine levels (r = 0.59), and PGE2 levels (r = 0.75), while showing a notable negative correlation with colonic transit time (r = −0.82), central/ peripheral area time ratio (r = −0.76), acetate (r = −0.52), propionate (r = −0.78), butyrate (r = −0.72) and the relative abundance of *Akkermansia* (r = −0.74) and *Lactobacillus* (r = −0.56). These results indicate a significant association between SCFAs, inflammation, and gut microbiota. Furthermore, a notable correlation was identified among histamine (r = 0.79), mast cell tryptase (r = 0.50), and PGE2 (r = 0.71) with AWR scores, indicating a strong association between mast cell hyperactivation and visceral hypersensitivity. The findings demonstrated that the ameliorative effect of synbiotic treatment on IBS was intricately associated with the composition and structure of the gut microbiota, the levels of fecal SCFAs, the integrity and functionality of the gut barrier, as well as the modulation of the inflammatory responses.

## 4. Discussion

In the present study, GOS was identified as the optimal carbon source for the utilization of *L. plantarum* ZYC501 and, therefore, establishing the synbiotic. Subsequently, *C. rodentium* was used to induce the mouse model of IBS-D, and the synbiotics of *L. plantarum* ZYC501 and GOS were introduced to investigate the effects on modulating IBS-associated intestinal perturbations and the underlying mechanisms. As expected, mice in the IBS group showed colonic transit dysfunction, visceral hypersensitivity, anxiety-like behavior, compromised mucosal integrity, alteration of gut microbiota composition, and mast cell hyperactivation compared to the mice in the CON group. Although the interventions with *L. plantarum* ZYC501, GOS, as well as the synbiotic all markedly enhanced the integrity of the gut barrier, upregulated expression of MUC2 in the colon, improved gut microbiota composition, increased production of SCFAs, and reduced mast cells hyperactivation, the synbiotic exerted greater benefits on counteracting some of these perturbations, particularly on colonic transit function, as well as mucosal integrity in the context of IBS.

Physiological changes, including colonic transit dysfunction and visceral hypersensitivity, contribute substantially to the distress experienced by IBS-D patients [1]. The reduced duration of food retention led to the development of unformed stools in patients with IBS-D who exhibited impaired colonic transit function [48]. Visceral hypersensitivity represents a pathophysiological hallmark among patients with IBS-D, suggesting amplification of pain signals and the perception of discomfort during normal physiological stimuli or even minimal mechanical distension, such as that induced by rectal balloon inflation [49]. Studies indicate that individuals with IBS frequently experience comorbid anxiety or depression [24,50]. In this study, a model integrating *C. rodentium* infection with exposure to WAS was employed, which effectively simulated the physiological and psychological pathogenesis of IBS. Nevertheless, given that the observed alleviation of abdominal pain in IBS-D attributed to probiotics is confined to the model induced by *C. rodentium* and chronic water avoidance stress, the efficacy of the synbiotic in addressing other models of IBS-D remains uncertain, highlighting the need for further validation in future investigations.

The hyperactivation of mast cells, resulting in an augmented synthesis of PGE2 through COX-2, represents a pivotal mechanism underlying the pathogenesis of visceral hypersensitivity in IBS [10]. Additionally, LPS has been identified as a critical mediator that can elicit the hyperactivation of mast cells within the mucosal environment [8]. Upon measurement of various characteristic indicators of visceral hypersensitivity, a significant elevation in the levels of histamine and mast cell tryptase within the colon was noted. Additionally, there was a marked increase in the release of PGE2. These results indicate that chronic exposure to WAS activates mast cells, which subsequently enhances histamine and mast cell tryptase activity and induces persistent intestinal nerve excitation, ultimately resulting in visceral hypersensitivity that is characterized by elevated levels of PGE2. In addition to the manifestations of low-grade inflammation and visceral hypersensitivity, significant alterations in the diversity and composition of the gut microbiota in the mice were also observed. The literature indicates that IL-6 levels in patients with IBS are significantly elevated compared to those in the CON group, particularly among individuals with IBS-D [51]. Additionally, *TRPV1* has been shown to be activated by histamine, resulting in heightened visceral sensitivity [52]. Notably, the successful establishment of this murine model resulted in symptoms, including inflammatory cell infiltration, elevated expression of pro-inflammatory cytokines, and compromised gut barrier function. These findings suggested LPS may elicit the hyperactivation of mast cells to upregulation of COX-2 expression, leading to an increase in PGE2 synthesis, thereby resulting in visceral hypersensitivity, accompanied by inflammatory responses.

An expanding body of evidence indicates that compromised gut barrier function significantly contributes to the onset and progression of IBS [53,54]. Notably, the mucus layer, primarily composed of mucins, serves to inhibit the proliferation of pathogenic bacteria and facilitates interaction with intestinal epithelial cells [55]. In this study, the integrity of the mucus layer was compromised in the IBS model compared to the CON, while reversed by synbiotic intervention. In the view that mucus is primarily constructed around the highly glycosylated mucin, and the key component of this mucus is MUC2, a heavily glycosylated glycoprotein, particularly abundant in O-glycans. Within the gut environment, goblet cells specialize in regulating glycosylation processes and synthesizing mucus [56]. Furthermore, the expression of MUC2 in the colons of mice was assessed using immunofluorescence staining. The expression of MUC2 in the IBS group was significantly lower than that in the CON group. The intervention of synbiotics improved MUC2 expression compared to the IBS group. Additionally, this study has identified that impairment of the mucus layer barrier constitutes a critical factor in the pathogenesis of IBS [9]. Therefore, this provides a partial explanation for how synbiotics may reduce mast cell hyperactivation induced by LPS in IBS mice by restoring the compromised mucus layer barrier. Furthermore, MUC2 represents a promising therapeutic target for ameliorating IBS pathophysiology. However, the lack of targeted knockdown experiments specifically focusing on MUC2 within the colonic tissue of IBS mouse models has presented an obstacle to the comprehensive clarification and identification of the precise molecular targets associated with MUC2 in the context of IBS. Notably, the potential mechanistic interplay between MUC2 and gut microbiota composition, as well as its regulatory effects on microbial-derived metabolites, still remains unresolved. The precise mechanistic pathways through which MUC2 exerts its mucosal protective or immunomodulatory functions in IBS pathogenesis warrant systematic investigation in future research.

Interestingly, it was observed that the levels of propionate and butyrate of the IBS mice were significantly lower than those in the CON group, while synbiotic intervention was able to restore these levels. This finding was consistent with previous research that reported lower concentrations of acetate and propionate in the feces of IBS-D patients [57]. Therefore, it is speculated that synbiotics may restore the integrity of the mucus layer by increasing MUC2 expression, influenced by the levels of propionate and butyrate in the context of IBS. Numerous studies have documented that the composition of the gut microbiota in IBS patients is disrupted, exhibiting significant differences when compared to healthy individuals [11,12]. The significant alteration of gut microbiota composition among the three groups, the CON, IBS, and IBS + SYN groups, was observed in this study. The relative abundances of *Helicobacter* and *Saccharibacteria* were found to be significantly elevated in the IBS group. Cohort studies have demonstrated a robust association between Helicobacter and both the onset and progression of IBS, indicating that the presence of *Helicobacter* may heighten the risk of developing this condition [58,59]. LPS derived from Helicobacter pylori was elucidated to provoke a cascade of immune responses [60]. It is postulated that LPS originating from Gram-negative bacteria, exemplified by *Helicobacter*, has the potential to activate mast cells within the mucosal layer, thereby instigating subsequent visceral hypersensitivity reactions. Moreover, it was discovered that the relative abundance of *Akkermansia* at the genus level was significantly lower in the IBS group compared to both the CON and IBS + SYN groups. It was reported that the gut barrier in the colon was compromised, and the relative abundance of *Akkermansia* was also reduced in colorectal cancer mice [13]. Furthermore, *Akkermansia* has been shown to stimulate the production of mucins [61], suggesting that the compromise of barrier integrity may be linked to mucin production. *Akkermansia* has been shown to produce SCFAs that promote mucin production, especially butyrate [62,63,64]. SCFAs are synthesized and accumulated within the colon, with acetate, propionate, and butyrate being the predominant SCFAs generated [65]. The SCFAs, which are primarily produced by the anaerobic fermentation of dietary fiber by the gut microbiota, play a crucial role in maintaining the integrity of the gut barrier. The mechanism by which SCFAs mediate the enhancement of gut barrier integrity involves multiple pathways [66]. Notably, propionate and butyrate increased the MUC2 expression of MUC2 mRNA, mediated via the acetylation/methylation of histones at the MUC2 promoter [67].

This study introduces a novel synbiotic formulation designed to alleviate IBS through a previously unexplored mechanistic pathway. Specifically, it is proposed that the synbiotic-mediated amelioration of gut microbiota dysbiosis enhances the structural and functional integrity of the intestinal mucus layer, thereby attenuating LPS-induced mast cell hyperactivation and subsequent visceral hypersensitivity. While the findings highlight the critical role of MUC2 in this process, it is important to note that the mucus layer is a complex ecosystem comprising antimicrobial peptides, immunoglobulins, and other bioactive molecules that collectively regulate pathogenic bacterial proliferation and modulate the production of inflammatory mediators, including LPS. Furthermore, although this investigation focused on mast cells as central effectors in IBS pathophysiology, the intestinal immune response is a multifaceted network involving diverse cell populations. For instance, macrophages, which are pivotal in maintaining intestinal immune homeostasis, may also contribute significantly to the inflammatory cascade observed in IBS. Their role in mediating cytokine release, antigen presentation, and tissue repair warrants systematic exploration in future studies to fully elucidate the immunoregulatory mechanisms underlying synbiotic efficacy.

## 5. Conclusions

This study demonstrates that supplementation with the synbiotic composed of *L. plantarum* ZYC501 and GOS significantly alleviates colonic transit dysfunction, visceral hypersensitivity, and anxiety-like behavior in IBS mice. The synbiotic treatment profoundly altered the composition of gut microbiota to reduce mast cell hyperactivation, thereby decreasing the expression of COX-2 as well as PGE2 and improving the integrity of the mucus layer by elevating SCFAs levels to elevate MUC2 expression. Therefore, these findings furnish a theoretical basis for microbiota-targeted therapeutic interventions to alleviate gut disorders through the modulation of gut microbiota, representing a promising alternative for a non-pharmacological therapeutic approach.

## Data Availability

The original contributions presented in the study are included in the article/Supplementary Materials. Further inquiries can be directed to the corresponding authors.

## Supplementary Materials

The following supporting information can be downloaded at: https://www.mdpi.com/article/10.3390/nu17101670/s1, Table S1: Compositions of AIN-93M diet; Table S2: Primer sequences used for RT-qPCR; Figure S1: Effects of different carbon sources on the growth curve of *L. plantarum* ZYC501. (A–D) Growth curves of *L. plantarum* ZYC501. at 5 g/L (A), 10 g/L (B), 20 g/L (C), 30 g/L (D); Figure S2: Effects of synbiotic on gut microbiota in IBS mice. (A) PCoA of CON, IBS and LP (IBS + L.p.) group; (B) PCoA of CON, IBS and GOS (IBS + GOS) group; (C) Display diagram of significantly different species with LDA score greater than 4.0 (CON, IBS and LP group) (n = 6); (D) Display diagram of significantly different species with LDA score greater than 4.0 (CON, IBS and GOS group) (*n* = 6). (E–H) Relative abundance of *Lactobacillus*, *Akkermansia*, *Helicobacteraceae*, and *Saccharibacteria* at the genus level (*n* = 6). Means with different letters (a, b, c) are significantly different from each other (*p <* 0.05).
