# Supplementary material for: Lactobacillus plantarum and Galacto-Oligosaccharides Synbiotic Relieve Irritable Bowel Syndrome by Reshaping Gut Microbiota and Attenuating Mast Cell Hyperactivation
Source: Nutrients. 2025 May 14;17(10):1670. doi: 10.3390/nu17101670 (PMC12113827; doi:10.3390/nu17101670)
Supplement: Supplementary file 1 [file nutrients-17-01670-s001.zip › nutrients-3627614-supplementary.pdf]

Supplementary Tables and Figures

Table S1 Compositions of AIN-93M diet

Classification	Content（g/kg）
Corn Starch	465.700
Maltodextrin	155.000
Casein	140.000
Sucrose	100.000
Cellulose	50.000
Soybean Oil	40.000
Mineral Mix, M1021	35.000
Vitamin Mix, V1010	10.000
L-Cystine	1.800
Choline chloride	2.500
Tert-Butylhydroquinone	0.036
Total (g)	1000.000

Table S2 Primer sequences used for RT-qPCR

Name	Forward Primers (5’-3’)	Reverse Primers (5’-3’)
IL-6	CTCTGGCGGAGCTATTGAGA	AAGTCTCCTGCGTGGAGAAA
TNF-α	CTCATGCACCACCATCAAGG	ACCTGACCACTCTCCCTTTG
TRPV1	CATCCTCCTGCTCAACATGC	GCCTTCCTCATGCACTTCAG
MUC2	GAAGCCAGATCCCGAAACCA	CCAGCTTGTGGGTGAGGTAG
GAPDH	TGGAGAAACCTGCCAAGTATGA	TGGAAGAATGGGAGTTGCTGT

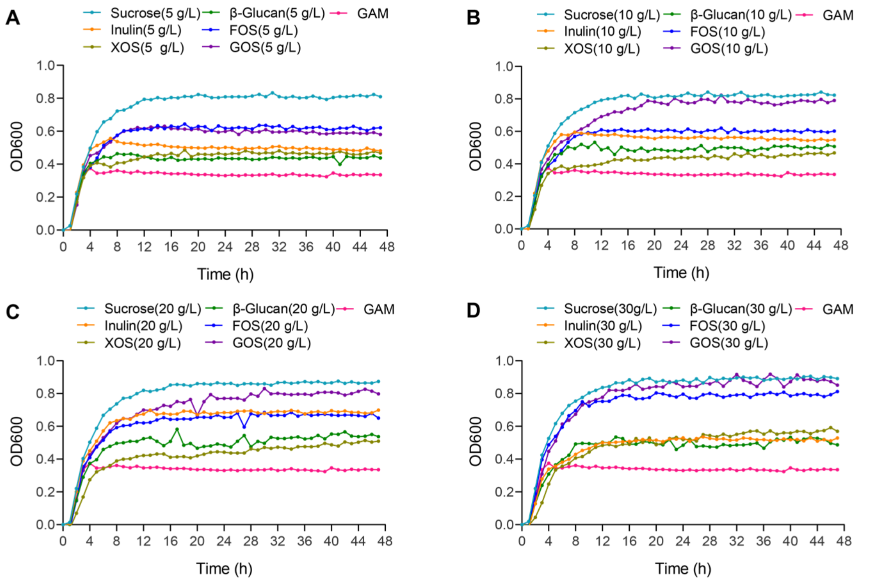

Figure S1 Effects of different carbon sources on the growth curve of *L. plantarum* ZYC501. (A-D) Growth curves of *L. plantarum* ZYC501 at 5 g/L(A), 10 g/L(B), 20 g/L(C), 30 g/L(D).

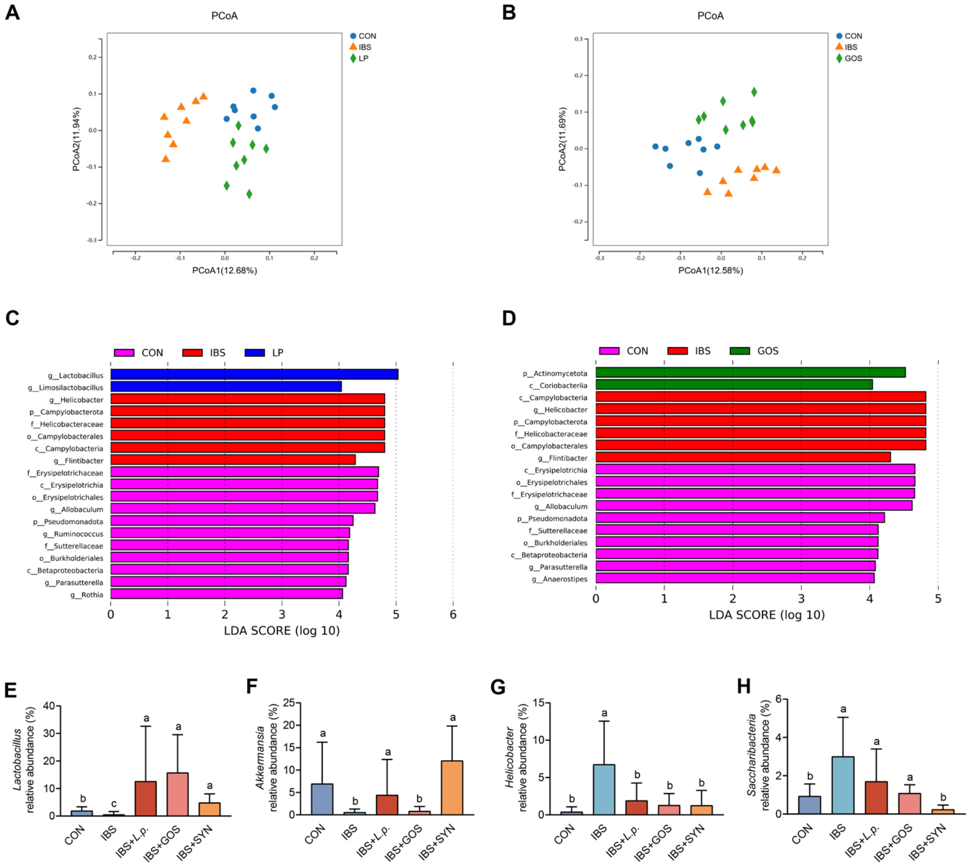

Figure S2 Effects of synbiotic on gut microbiota in IBS mice. (A) PCoA of CON, IBS and LP (IBS + *L. p.*) group; (B) PCoA of CON, IBS and GOS (IBS + GOS) group; (C) Display diagram of significantly different species with LDA score greater than 4.0 (CON, IBS and LP group) (n = 6); (D) Display diagram of significantly different species with LDA score greater than 4.0 (CON, IBS and GOS group) (n = 6). (E-H) Relative abundance of *Lactobacillus*, *Akkermansia*,

Helicobacteraceae, and *Saccharibacteria* at the genus level (n = 6). Means with different letters (a, b, c) are significantly different from each other ($p < 0.05$).
